# Persistent Trichomoniasis in Pregnancy: A Case Report Calling for Further Research to Alternative Antibiotic Treatment

**DOI:** 10.7759/cureus.80702

**Published:** 2025-03-17

**Authors:** Donya T Sabet, Hainian Yu, Luke Prior, Lilantha Wedisinghe

**Affiliations:** 1 Obstetrics and Gynecology, Ipswich Hospital, Brisbane, AUS; 2 Faculty of Medicine, Griffith University, Gold Coast, AUS; 3 School of Medicine, Bond University, Gold Coast, AUS; 4 Internal Medicine, Ipswich Hospital, Brisbane, AUS

**Keywords:** metronidazole resistance, metronidazole-resistant trichomoniasis, persistent trichomoniasis, preterm birth, recurrent trichomoniasis infection, sexually transmitted diseases, threatened preterm labor, trichomonas vaginalis, trichomoniasis, trichomoniasis in pregnancy

## Abstract

Trichomoniasis is an STI caused by a microscopic parasite known as *Trichomonas vaginalis*. It is considered one of the most widespread nonviral STDs globally. Trichomoniasis during pregnancy can lead to complications such as early rupture of membranes and premature birth. The standard treatment for trichomoniasis in adults, including pregnant individuals, is metronidazole. Recurrent cases may occur due to factors such as incomplete treatment, reinfection, or metronidazole resistance. In cases of persistent trichomoniasis where noncompliance and reinfection have been ruled out, metronidazole resistance should be considered. We present the case of a 39-year-old woman, gravida 4, para 3, with persistent trichomoniasis unresponsive to multiple courses of metronidazole, suggesting potential metronidazole resistance. She experienced recurrent symptoms of threatened preterm labor, likely due to uterine irritability caused by trichomoniasis. While various treatment regimens are available for metronidazole resistance, their safety profiles have not been evaluated during pregnancy. Further research is essential to identify a safe and effective treatment for metronidazole-resistant trichomoniasis in pregnancy.

## Introduction

Trichomoniasis, caused by the protozoan *Trichomonas vaginalis*, is the most common nonviral STD worldwide [1], with an estimated prevalence of approximately 1% in Australia [2]. While it can be asymptomatic, symptoms may include frothy, yellow-green vaginal discharge, vulvar itching, and discomfort. During pregnancy, trichomoniasis is associated with complications such as preterm rupture of membranes and preterm birth [3], making prompt diagnosis and treatment essential.

Metronidazole is the recommended first-line treatment for trichomoniasis in all adults, including pregnant individuals. However, recurrent trichomoniasis can occur due to noncompliance, reinfection, or treatment failure related to metronidazole resistance [4]. Although metronidazole resistance in persistent trichomoniasis is well documented, evidence-based recommendations for second-line treatment are lacking.

We present a case of persistent trichomoniasis during pregnancy complicated by uterine irritability. Despite multiple courses of metronidazole treatment, the infection persisted throughout pregnancy. This case underscores the need for further research to identify effective alternative antibiotics for treating metronidazole-resistant trichomoniasis.

## Case presentation

A 39-year-old woman, gravida 4, para 3, presented at the antenatal clinic at 21+1 weeks gestation for a routine visit. She had a history of persistent trichomoniasis lasting over two years, despite receiving four courses of metronidazole. She was in a monogamous, long-term relationship with a partner who had consistently tested negative for *T. vaginalis *and had undergone prophylactic treatment. She had last tested positive for trichomonas during her first antenatal visit with her family doctor, at which time she was treated with a course of metronidazole. At her 21+1 week visit, a test of cure was collected along with a screen for chlamydia and gonorrhea. The result returned positive for trichomonas (Table 1). A presumptive diagnosis of persistent trichomoniasis was made. After consulting with the obstetric medicine team, a routine course of 400 mg oral metronidazole twice daily for seven days was recommended, followed by a repeat test of cure after one month. She was advised to have her partner tested and treated if necessary and to remain abstinent until both partners had tested negative.

**Table 1:** STI screening results

Gestational age at date of testing	*Trichomonas vaginalis* DNA	*Chlamydia trachomatis* DNA	*Neisseria gonorrhoeae* DNA
21+1	Detected	Not detected	Not detected
28+1	Detected	Not tested	Not tested
33+5	Detected	Not detected	Not detected
Four days postpartum	Detected	Not detected	Not detected

**Figure 1:**
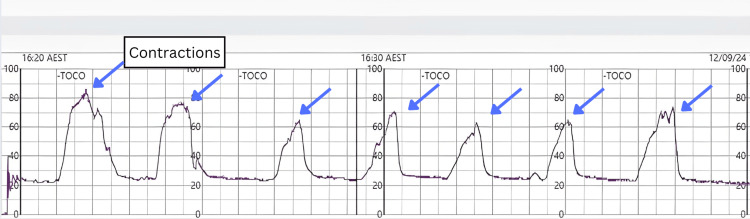
Tocodynamometer demonstrating uterine contractions Tocodynamometer demonstrating uterine contractions (blue arrows) detected every two to three minutes.

At 24+4 weeks gestation, she presented to the maternity day assessment unit with signs of threatened preterm labor (TPTL). She reported experiencing daily intermittent lower abdominal cramping for the past week. Upon assessment, she was found to be contracting every two to three minutes with moderate intensity, each lasting 45-50 seconds, as confirmed through palpation and a tocodynamometer (Figure 1). A speculum examination revealed a long, closed cervix, and a test to exclude preterm labor was negative. A recent routine ultrasound, performed three days prior, showed a normal cervical length of 45 mm (normal >35 mm). Despite receiving analgesia and nifedipine according to protocol, her contractions persisted, leading to her transfer to a tertiary center. She was diagnosed with uterine irritability secondary to trichomoniasis. A course of metronidazole was reinitiated, and she was discharged once her contractions settled.

She returned for her follow-up appointment at 28 weeks, at which point her test of cure again returned positive for trichomonas (Table 1). After consultation with the infectious diseases team, a further seven-day course of metronidazole was recommended, with the same precautions as before. Currently, routine clinical testing for metronidazole resistance in *T. vaginalis* is not available. As a result, a presumptive diagnosis of metronidazole-resistant trichomoniasis was made. This led to a multidisciplinary team recommendation to avoid further treatment with metronidazole, except for a final course one week before her elective repeat cesarean section.

She presented to the maternity day assessment unit on four additional occasions between 29 and 36+6 weeks of gestation, each time showing signs of TPTL. During each presentation, she experienced regular, moderate-intensity contractions that required opioid analgesia. At her final presentation, at 36+6 weeks, she reported contractions and per vaginal spotting. Cardiotocography showed signs of fetal distress, prompting an emergency cesarean section. A male infant weighing 2,640 g was delivered. He was assessed by pediatricians as being in good condition, and antibiotic therapy was not indicated.

## Discussion

The identification and treatment of trichomoniasis during pregnancy is crucial due to its potential to cause adverse birth outcomes. A 2021 systematic review found significant associations between trichomoniasis during pregnancy and prelabor rupture of membranes, preterm delivery, and low birth weight [3].

Treatment options for trichomoniasis are limited, as nitroimidazoles are the only class of antimicrobials known to be effective against *T. vaginalis *[5,6]. The standard first-line treatment is a seven-day course of oral metronidazole 400 mg twice a day, which is recommended for all cases, including during pregnancy [7].

In cases of persistent trichomoniasis, factors such as patient noncompliance, reinfection, and metronidazole resistance should be considered. When compliance is a concern, an alternative regimen of single-dose oral metronidazole 2 g has shown significantly better compliance rates [8]. However, due to the theoretical risk of teratogenicity with higher doses of metronidazole during pregnancy, there is no consensus on its recommendation [9]. To prevent reinfection, patients should be advised to treat their sexual partner(s) simultaneously and abstain from intercourse until their partner(s) have completed treatment and follow-up [9].

If trichomoniasis persists despite addressing noncompliance and reinfection, metronidazole resistance should be suspected. Metronidazole resistance occurs in 4-10% of cases of vaginal trichomoniasis [10]. Several alternative regimens have been trialed, including a seven-day course of high-dose metronidazole 2 g orally daily or a 14-day course of oral tinidazole 2 g daily, with concurrent intravaginal tinidazole 500 mg twice daily [6,11]. Although some case studies have reported successful use of tinidazole in pregnant patients with persistent trichomoniasis, data on its safety during pregnancy is limited [12-14]. Currently, there are no evidence-based second-line recommendations for persistent or resistant infections during pregnancy and no data to guide the treatment of sexual partners of pregnant women with treatment failure [9].

This case report highlights the treatment challenges associated with persistent trichomoniasis during pregnancy. Further research is needed to identify alternative therapies with an evidence-based safety profile for treating metronidazole-resistant cases.

Additionally, this case emphasizes the impact of antibiotic prescribing practices on metronidazole resistance. A 2023 retrospective study analyzing Hospital National Antimicrobial Prescribing Survey data from 2013 to 2021 found that metronidazole was the fifth most prescribed antimicrobial, with 46.5% of prescriptions not in compliance with prescribing guidelines [15]. Quality improvement initiatives are essential to refine metronidazole prescribing practices in order to reduce resistance rates.

## Conclusions

Trichomoniasis during pregnancy is linked to adverse birth outcomes, and metronidazole is the first-line treatment. In cases of persistent trichomoniasis, metronidazole resistance should be considered. However, the safety profile of current treatment options for metronidazole-resistant trichomoniasis has not been studied during pregnancy, and there is no consensus on their use. Further research is essential to identify a safe and effective treatment for metronidazole-resistant trichomoniasis in pregnancy.
